# Effects of abiraterone acetate plus prednisone on bone turnover markers in chemotherapy-naïve mCRPC patients after ADT failure: A prospective analysis of the italian real-world study ABITUDE

**DOI:** 10.1016/j.jbo.2020.100341

**Published:** 2020-12-02

**Authors:** Daniele Santini, Saverio Cinieri, Donatello Gasparro, Roberto Bordonaro, Pamela Francesca Guglielmini, Vincenzo Emanuele Chiuri, Rolando M D'Angelillo, Giovanni Luca Ceresoli, Daniele Fagnani, Mirko Acquati, Manlio Mencoboni, Gaetano Lanzetta, Donata Sartori, Paolo Carlini, Fabiana Panebianco, Patrizia Beccaglia, Giuseppe Procopio

**Affiliations:** a Department of Oncology, Campus Bio-Medico University, Rome, Italy; b Medical Oncology Unit, Antonio Perrino Hospital, Brindisi, Italy; c Medical Oncology Unit, Department of General & Specialistic Medicine, University Hospital of Parma, Italy; d MD – ARNAS Garibaldi, Catania, Italy; e Medical Oncology Department, Ospedale SS Antonio e Biagio e Cesare Arrigo, Alessandria, Italy; f Medical Oncology Department, Ospedale Vito Fazzi, Lecce, Italy; g Department of Biomedicine and Prevention, Tor Vergata University, Rome, Italy; h Department of Oncology, Cliniche Humanitas Gavazzeni, Bergamo, Italy; i Medical Oncology Division – ASST, Vimercate, Italy; j Unit of Medical Oncology, Azienda Ospedaliera San Gerardo, Monza, Italy; k Oncology Unit, Villa Scassi Hospital, 16149 Genova, Italy; l Department Oncology and Palliative Care, INI Grottaferrata, Rome, Italy; m Oncology Unit, AULSS 3, Mirano, Italy; n Division of Medical Oncology 1, IRCCS “Regina Elena” National Cancer Institute, Rome, Italy; o Medical Affairs Department, Oncology, Janssen-Cilag SpA, Cologno Monzese, Milan, Italy; p Department of Medical Oncology, Fondazione IRCCS, Istituto Nazionale dei Tumori, Milano, Italy

**Keywords:** Abiraterone acetate, Bone alkaline phosphatase, Bone targeting therapy, Bone turnover biomarkers, C-terminal telopeptide, mCRPC

## Abstract

**Background:**

Bone remodeling is disrupted in metastatic disease, which affects > 70% of metastatic castration-resistant prostate cancer (mCRPC) patients. As a result, abnormal levels of specific bone turnover biomarkers (BTMs) are released. In this prospective ancillary analysis of the Italian real-world study ABITUDE, four markers were measured during abiraterone acetate plus prednisone (AAP) treatment in chemotherapy-naïve mCRPC men failing androgen-deprivation therapy.

**Methods:**

Patients were enrolled if a blood sample was obtained before the first administration of abiraterone (baseline); ad-hoc blood samples were withdrawn during routine tests after 3, 6, and 12 months. A centralized lab measured bone alkaline phosphatase (BALP, osteoblast activity marker), type-I collagen-C-telopeptide (CTX-1, bone resorption marker), parathyroid hormone (PTH) and vitamin D (vitD). At each time point, intra-patient variations vs baseline were compared by the signed-rank test (statistical significance: P-value < 0.05).

**Results:**

Of 481 patients enrolled in ABITUDE, 186 (median age: 76 [range: 53–93] years) met the substudy criteria: 74.7% had bone metastases, 11.8% were on bone-targeted therapies (BTT) and 14.0% on vitD supplementation. BALP decreased significantly at month 6 (P = 0.0010) and 12 (P < 0.0001) and CTX-1 at month 6 (P = 0.0028); PTH increased at month 3 (P < 0.0001); no significant difference in vitD levels was observed. Similar findings were observed in BTT-untreated patients. The reduction in BALP and CTX-1 levels was more pronounced in patients with than without bone metastases; in the latter group, no significant variation in BALP and CTX-1 levels was observed.

**Conclusions:**

AAP seems to exert an effect on the microenvironment of metastatic but not of normal bone, which likely contributes to its antitumoral activity.

## Highlights

• Bone remodeling is disrupted in metastatic disease, affecting > 70% of mCRPC men.
• In metastatic disease, abnormal levels of specific BTMs are released.
• We prospectively measured four BTMs markers in chemotherapy-naïve mCRPC men on AAP therapy.
• AAP seems to act on the microenvironment of metastatic but not of normal bone.
• This action likely contributes to the antitumoral activity of AAP.

## 1 Introduction

Bone remodeling is a dynamic process that ensures the maintenance of skeletal integrity [1]. The balance between bone apposition and resorption is disrupted in prostate cancer (PCa) as a consequence of the high tropism of PCa cells for the bone. The complex crosstalk with the microenvironment [2], [3], [4] leads to a “vicious cycle” that favors the growth of bone metastases, found in >90% of patients throughout the disease course [5] and in >70% of those with metastatic castration-resistant PCa (mCRPC) [6], and culminating in substantial morbidity and mortality. In particular, bony disease is associated with an increased incidence of skeletal-related events (SREs) [7], [8] (overall incidence rate: 3.78 [95% confidence interval 3.53–4.03] per 100 person-months) [7].

By imaging, PCa-derived bone metastases appear predominantly osteoblastic; increased osteoblastic activity induces secondary hyperparathyroidism (observed in 21% to 57% of patients with advanced PCa [9], [10], [11]), that, in turn, promotes osteoclast activation at distant sites to stimulate calcium release. Indeed, osteoblastic lesions frequently present also an osteolytic pattern that may extend beyond the sites of metastases and is responsible for the occurrence of SREs.

During bone remodeling, bone-associated proteins and mineral components are released into the bloodstream and urine; since their levels are altered in metastatic bone disease, several attempts have been made to use such bone turnover markers (BTMs) as diagnostic and prognostic tools in bone-dominant mCRPC [2], [12], [13], [14], [15], [16], [17], [18]. These include 1) bone alkaline phosphatase (BALP), which is reflective of both osteoblastic activity and disease extent (i.e., volume of metastases), and whose elevated levels at baseline correlate with worst outcomes; 2) C-telopeptide of type 1 collagen (CTX-1), a marker of resorption occurring both in metastatic and normal bone, whose levels are increased in mCRPC patients. Other bone-related markers frequently measured are 1) parathyroid hormone (PTH), a marker of osteoblastic activity and a major determinant of bone resorption and of PCa cell proliferation and migration, whose levels can be decreased by bone-targeted therapies (BTT) and by vitamin D (vitD) supplementation; 2) vitD, frequently deficient in elderly males, that enhances bone resorption to increase calcium bioavailability [18], [19], [20], [21], [22], [23]. Identifying reliable markers would be important in clinical practice also to possibly inform decisions and monitor response to therapy. However, at present, data are scarce [14], [16] and do not allow the routine use of BTMs.

In the expanding therapeutic landscape of mCRPC [24], abiraterone acetate (AA) is a prodrug for abiraterone, which is the first in class steroid 17alpha-hydrolase/C17,20 lyase complex inhibitor, that suppresses testosterone production by testes, adrenals and tumor cells to castrate-range levels. Besides, abiraterone inhibits the synthesis of dihydrotestosterone (the androgen receptor’s active ligand) from precursor steroids. In combination with prednisone (AAP), it has been approved to treat metastatic PCa patients [25], [26], [27]. In the COU-AA-302 trial, conducted in chemotherapy (CT)-naïve mCRPC men, AAP yielded a significant prolongation of radiographic progression-free survival, overall survival, and a significant delay in clinical decline, pain and CT initiation compared to placebo [25], [26], [28]. However, the trial did not include bone-related endpoints, such as the time to SREs and assessment of BTM levels. This benefit was subsequently confirmed in retrospective real-world studies [29], [30] and in the large Italian multicenter, prospective observational study ABITUDE [31]. Indeed, in line with COU-AA-302, the latter reported a 1-year probability of no radiographic progression of 73.9%, together with a significant reduction in mean and worst pain intensity and a significant improvement in daily activity interference after 6 months of therapy in symptomatic patients [31]. Moreover, ABITUDE prospectively assessed for the first time the levels of specific BTMs and bone-related markers during AAP treatment in a real-world setting: the fluctuations of BALP, CTX-1, PTH and vitD during the first year of patient observation under AAP therapy are described in the present ancillary analysis.

## 2 Materials and methods

### 2.1 Study design and patient population

This is an ancillary study of ABITUDE, a large Italian multicenter prospective cohort study evaluating the effectiveness of AAP in CT-naïve mCRPC patients who had failed ADT and in whom CT was not clinically indicated. Patients were enrolled over a 16-month period [32], at the start of AAP therapy, which had to occur within 30 days of the baseline visit, and were followed up to the end of observation, death or voluntary study withdrawal (whichever occurred first) [32]. AAP was given as per clinical practice. All patients were already on luteinizing hormone releasing hormone (LH-RH) agonists at the time of AAP commencement.

ABITUDE was approved by the ethic committees of the participating centers and was conducted according to the principles of the Declaration of Helsinki and the Good Clinical Practice guidelines. All patients provided written informed consent to participate in the study.

The present analysis was approved by each Center’s ethic committee, but the coordinator was the National Cancer Center of Milan (Code: 212082PCR4034; approval date: 22 Sep 2015). Participation was voluntary and eligible subjects signed a dedicated form to consent to the collection of an additional vial of blood (without the need of an extra puncture) during routine exams performed at the site. Undergoing blood withdrawal before the first administration of AAP was mandatory to participate in this analysis.

### 2.2 Data collection and assessment of bone turnover biomarkers

Before the start of AAP therapy, demographics and clinical information was retrieved from medical records. The following data were included in the present analysis for evaluable patients: age, body mass index (BMI), relevant medical history, historical data on PCa and metastatic disease (i.e. date of diagnosis and of castration, Gleason score at diagnosis, prostate-specific antigen [PSA], Eastern Cooperative Oncology Group-performance status [ECOG-PS], Tumor-Node-Metastasis [TNM] stage, metastasis site(s) and number of bone metastases). In addition, data about other relevant drugs administered during the study were collected.

The following BTMs and bone-related markers were assessed before the start of AAP therapy (baseline) and at month 3, 6, and 12 of therapy: CTX-1, PTH, vitamin D and BALP. An ad-hoc blood sample (1 vial, 5 ml) was withdrawn during scheduled routine blood tests at each site; due to fluctuations in the BTM levels (especially in the case of CTX-1), it was recommended to collect blood samples in the early morning and repeat withdrawals at the same time of the day during the subsequent visits. At each time point, only patients that were still on AAP treatment were included in the analyses.

Serum (for quantitation of BALP, CTX-1 and vitD) and plasma (for PTH evaluation) were prepared from blood samples, aliquoted and stored at −20 °C, to be subsequently shipped to the centralized lab (at S. Raffaele Hospital, Milan, Italy) for testing. The following kits were used, according to the manufacturer’s instructions: chemiluminescent immunometric automated assay (CLIA) supplied by DiaSorin (Stillwater, MN, USA) for BALP; ECLIA immunoassay (Roche) for PTH; ECLIA binding assay (Roche) for vitD; ECLIA sandwich-type immunoassay (Roche) for CTX-1. The reference intervals are as follows: 5.5–22.9 µg/L for BALP; 15–65 pg/mL for PTH; 20–68 ng/mL for vitD; 130–600 ng/L for CTX-1 in case of men aged 40 to 60 years and 100–600 ng/L for men aged > 60 years [33].

Evaluable patients with CTX-1 value < 0.05 ng/mL were excluded (N = 42) from this analysis as such values are below the level of detection of the method employed. Also evaluable patients with PTH value > 200 pg/mL (i.e. outliers) and evaluable patients with BALP value > 300 µg/L (i.e. outliers) were excluded (N = 7 and N = 12, respectively).

Beside reporting the raw values of each biomarker’ levels at baseline and at month 3, 6 and 12, due to the high number of drop-outs, data are also presented as the median intra-patient change recorded at every time point vs baseline, resulting from the comparison of paired samples at each time point, for every biomarker.

### 2.3 Statistical analysis

Continuous variables were summarized by descriptive statistics, and categorical variables by frequencies. The Wilcoxon signed-rank test was used to compare the median intra-patient change at month 3, 6 and 12 vs baseline for each biomarker. A P-value < 0.05 was considered statistically significant.

Data analysis were performed using SAS Enterprise Guide v.7.1 and SAS 9.4.

## 3 Results

### 3.1 Patients

Of the 481 patients enrolled in ABITUDE from February 2016 to June 2017, 202 from 29 Centers were included in this ancillary study, 186 of whom (92.1%) were evaluable: the most common reason was the lack of blood withdrawal before the first AAP administration (N = 16 [7.9%]).

The main demographic and clinical characteristics at baseline are summarized in Table 1. Briefly, the median age at enrollment was 76 (range: 53–93) years, and 108 (58.1%) patients were aged ≥ 75 years. At diagnosis, 89 (55.3%) patients had a Gleason score ≥ 8; 168 (90.3%) had undergone medical castration and 18 (9.7%) surgical castration. The duration of hormone-sensitivity period (calculated as the difference between the date of the first diagnosis of mCRPC and the date of castration) was ≥ 24 months in 97 (52.2%) men. 139 (74.7%) patients had bone metastases (bone metastases only in 132) and 47 (25.3%) had metastases in sites other than the bone. The median duration of the observation was 11.4 months (range 0.2–31.0).

**Table 1:** Demographic and clinical characteristics of evaluable patients (N = 186) recorded before the start of AAP therapy.

Characteristic	Evaluable patients N = 186
Age, median (range), years	76.0 (53.0–93.0)
BMI, median (range)	26.3 (18.6–42.2)
PSA, median (range), ng/ml	13.9 (0.1– 2779.1)

Gleason Score at tumor diagnosis, N (%)	
<8	72 (44.7)
≥8	89 (55.3)
missing	25 (13.4)

Duration of hormone-sensitivity period*, N (%)	
0–12 months	74 (48.7)
12–24 months	12 (7.9)
≥24 months	66 (43.4)

ECOG-PS, N (%)	
0	114 (62)
1	66 (35.9)
≥2	4 (2.2)
Missing	2 (1.2)

Extent of disease, N (%)	
Bones	139 (74.7)
Lymph nodes	96 (51.6)
Visceral	17 (9.1)
Other	7 (3.8)

Number of bone metastases	
1–3	45 (32.4)
4–9	44 (31.7)
≥10	30 (21.6)
Missing	20 (14.4)

Patients with ≥ 1 relevant pharmacological therapy administered during the study, N (%)	
Bone-Targeted Therapy	22 (11.8)
Denosumab	6 (3.2)
Bisphosphonates	16 (8.6)
- Zoledronic acid	15 (8.1)
- Alendronate	1 (0.5)
VitD supplementation	26 (13.9)
Calcium supplementation	7 (3.8)

*Calculated as difference between date of first diagnosis of mCRPC and date of castration.

BMI, body mass index; PSA, prostate-specific antigen; PCa, prostate cancer; mCRPC, metastatic castration-resistant prostate cancer; AAP, abiraterone acetate plus prednisone; CNS: central nervous system; ECOG-PS: Eastern Cooperative Oncology Group performance status; vitD, vitamin D.

Among the relevant therapies administered during the study there were BTT, given to 22 (11.8%) patients (ZA to 15, DNB to 6 and alendronate to 1), vitD supplementation to 26 (13.9%) and calcium supplementation to 7 (3.8%). Of those receiving BTT, 9 had bone metastases at baseline.

## 4 Assessment of bone turnover biomarkers

**Fig. 1:**
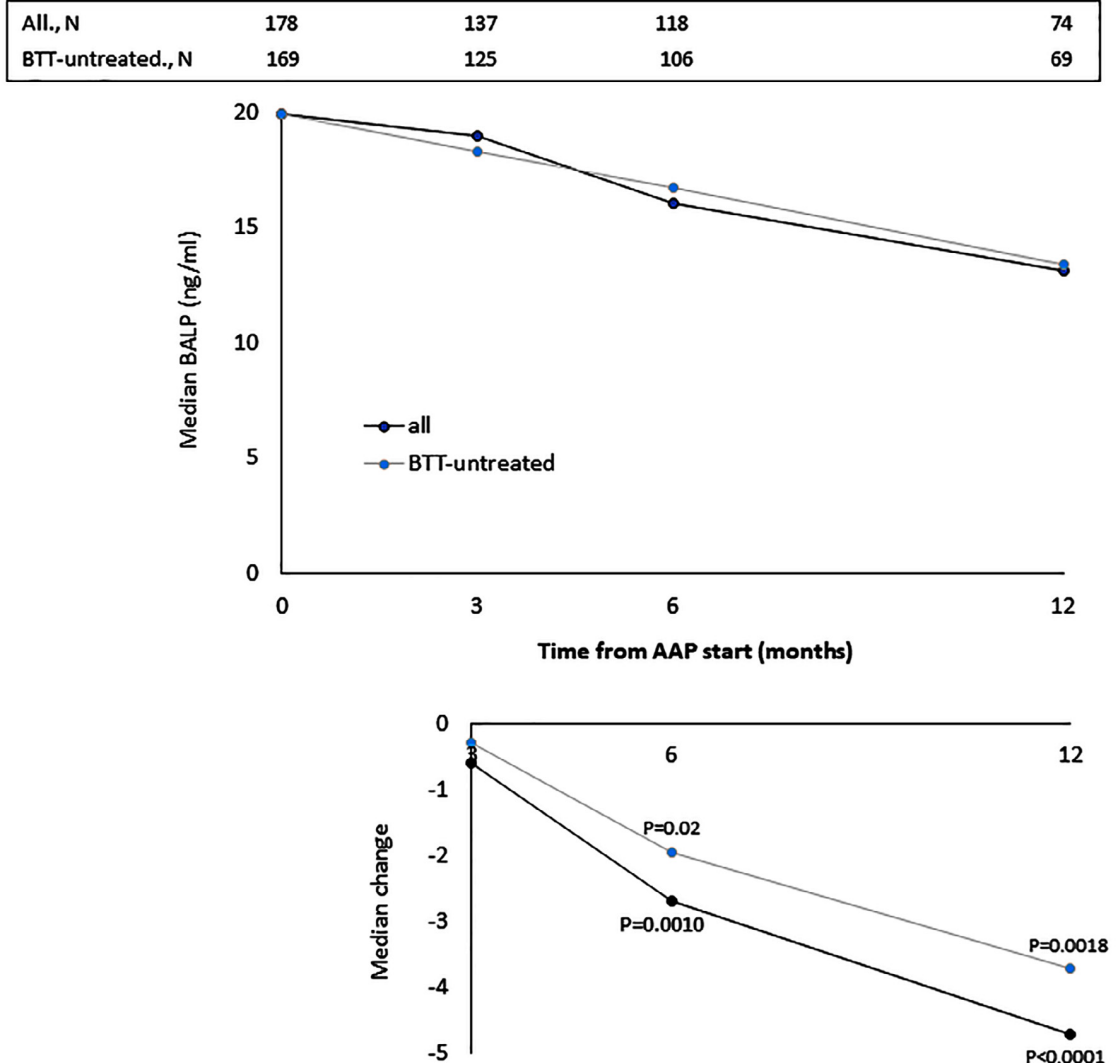
Evaluation of bone turnover biomarkers over time in the overall population and BTT-treated patients. Both the median levels (top panels) and the median change at 3, 6 and 12 months versus baseline (bottom panels) are reported for each biomarker. The Wilcoxon signed-rank test was used to compare the median change at month 3, 6 and 12 vs baseline for each biomarker (i.e. intra-patient change). The asterisks indicate statistical significance for the median change vs baseline.

**Fig. 2:**
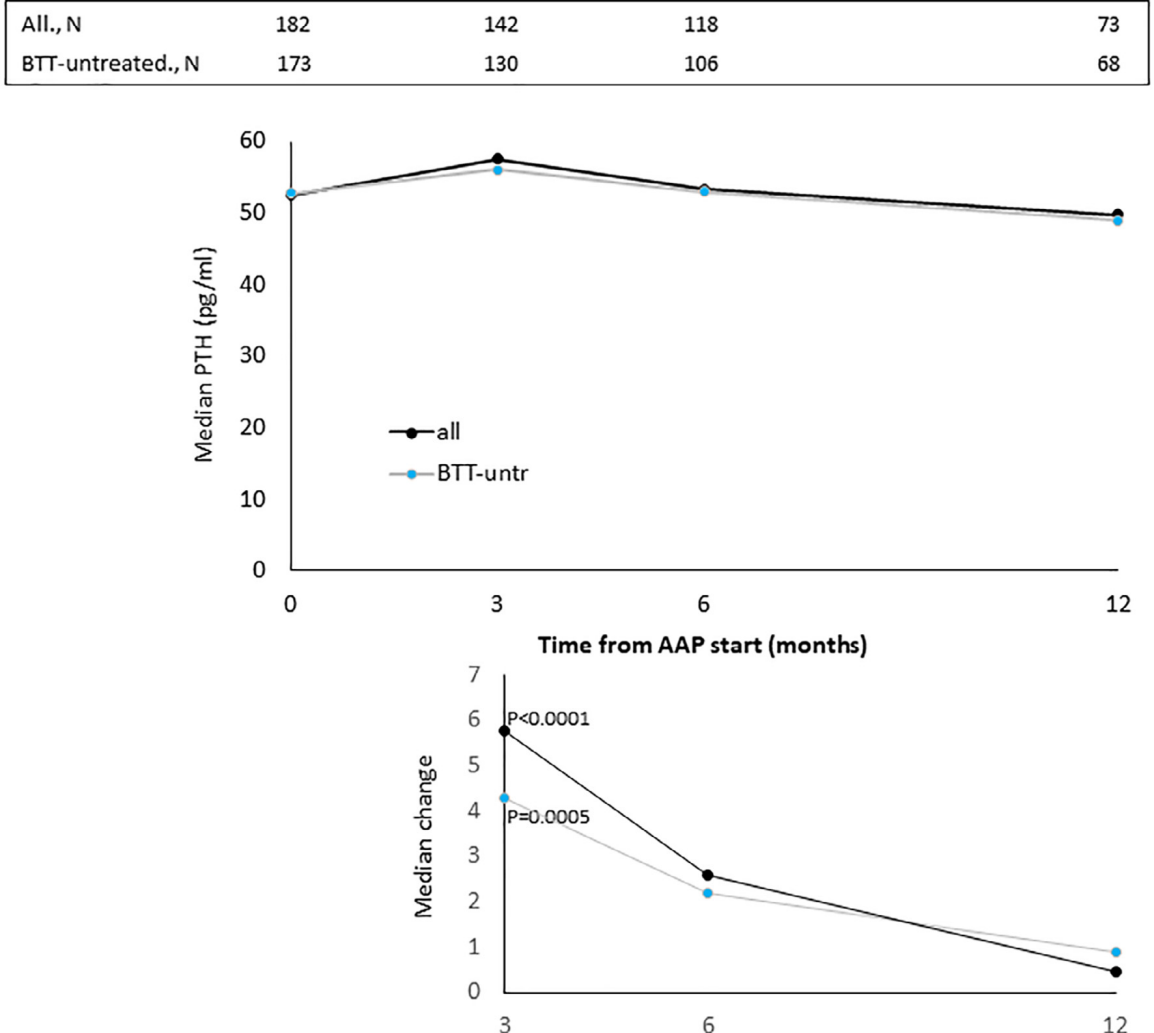
Evaluation of bone turnover biomarkers over time in the overall population and BTT-treated patients. Both the median levels (top panels) and the median change at 3, 6 and 12 months versus baseline (bottom panels) are reported for each biomarker. The Wilcoxon signed-rank test was used to compare the median change at month 3, 6 and 12 vs baseline for each biomarker (i.e. intra-patient change). The asterisks indicate statistical significance for the median change vs baseline.

**Fig. 3:**
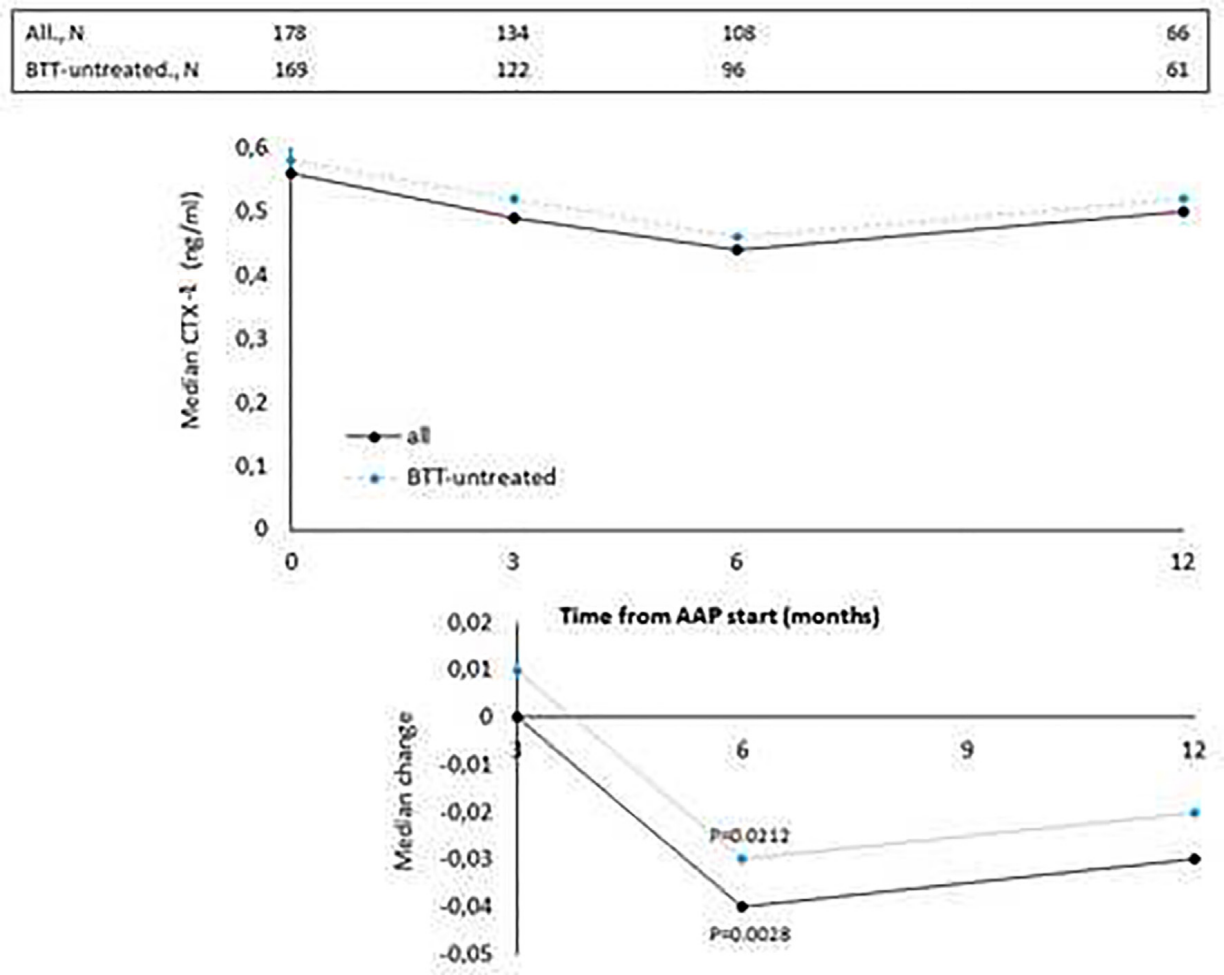
Evaluation of bone turnover biomarkers over time in the overall population and BTT-treated patients. Both the median levels (top panels) and the median change at 3, 6 and 12 months versus baseline (bottom panels) are reported for each biomarker. The Wilcoxon signed-rank test was used to compare the median change at month 3, 6 and 12 vs baseline for each biomarker (i.e. intra-patient change). The asterisks indicate statistical significance for the median change vs baseline.

**Fig. 4:**
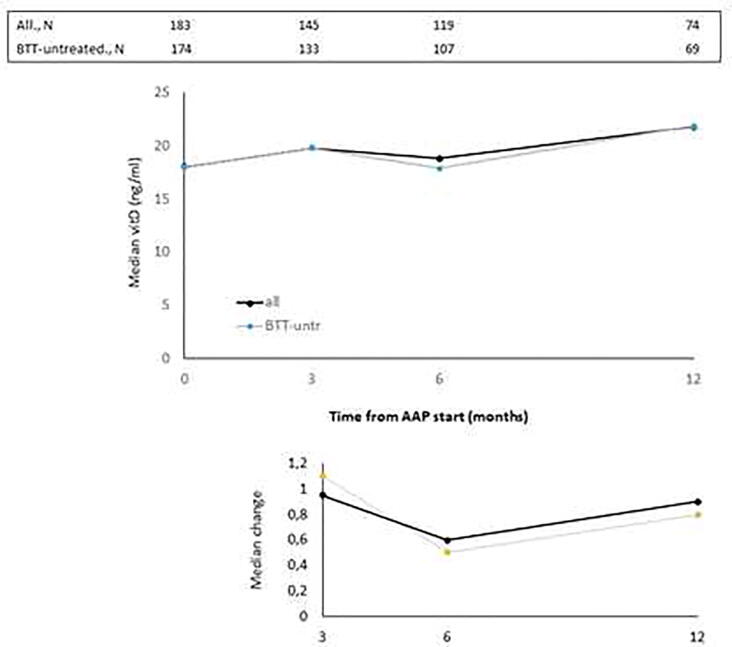
Evaluation of bone turnover biomarkers over time in the overall population and BTT-treated patients. Both the median levels (top panels) and the median change at 3, 6 and 12 months versus baseline (bottom panels) are reported for each biomarker. The Wilcoxon signed-rank test was used to compare the median change at month 3, 6 and 12 vs baseline for each biomarker (i.e. intra-patient change). The asterisks indicate statistical significance for the median change vs baseline.

The median values of BALP, CTX-1, PTH and vitD recorded at baseline and at month 3, 6 and 12 of AAP treatment are reported in Fig. 1, Fig. 2, Fig. 3, Fig. 4, top panels for both the overall population and BTT-untreated patients; the corresponding intra-patient changes are illustrated in Fig. 1, Fig. 2, Fig. 3, Fig. 4, bottom panels. The number of BTT-treated patients at each time point was extremely low (N = 9 at baseline, N = 12 at 3 months, N = 11 at 6 months and N = 5 at 12 months). BALP progressively decreased over time, with the change vs baseline becoming significant at month 6 and 12 in the overall population (P = 0.0010 and P < 0.0001, respectively) and in BTT-untreated men (P = 0.02 and P = 0.0018, respectively) (Fig. 1). PTH significantly increased at month 3 (all: P < 0.0001; BTT-untreated: P = 0.0005) and decreased thereafter (Fig. 2). CTX-1 progressively decreased until month 6, when the change was significant (all: P = 0.0028; BTT-untreated: P = 0.0212), and increased again at month 12 (Fig. 3). Finally, the levels of vitD fluctuated and no significant difference vs baseline was observed (Fig. 4).

### 4.1 Bone turnover biomarkers in men with vs without bone metastases

**Fig. 5:**
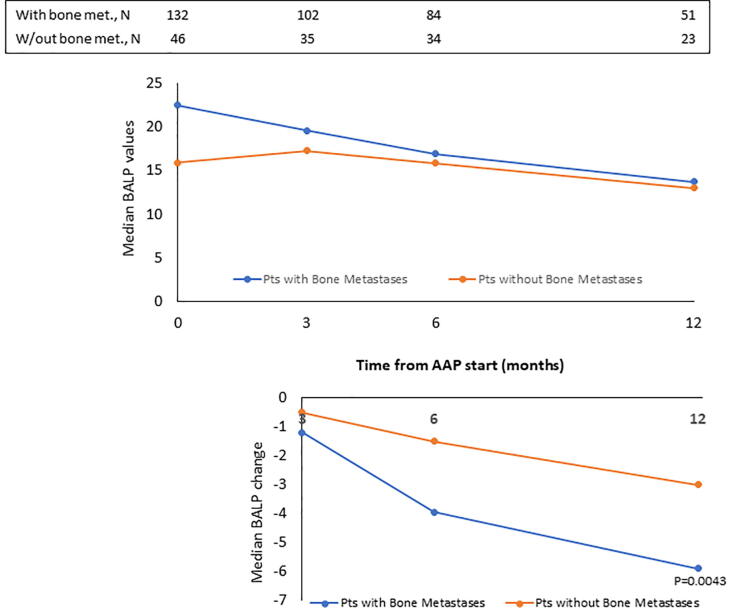
Evaluation of BALP levels over time in patients subgrouped by the presence of bone metastases. Top panel: median levels recorded; bottom panel: median intra-patient change at each time point vs baseline. The asterisk indicates statistical significance (P = 0.0234) reached at month 12 vs baseline in men with bone metastases only.

**Fig. 6:**
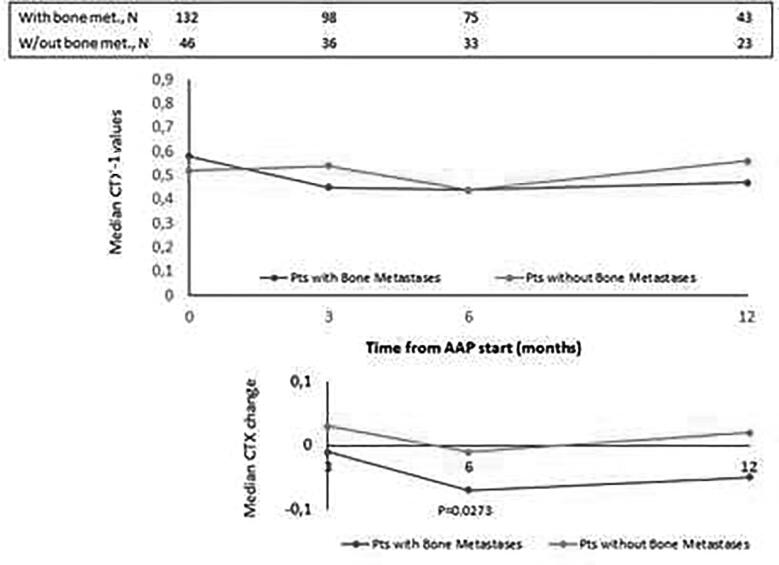
Evaluation of CTX levels over time in patients subgrouped by the presence of bone metastases. Top panel: median levels recorded; bottom panel: median intra-patient change at each time point vs baseline. The asterisk indicates statistical significance (P = 0.0072) reached at month 6 vs baseline in men with bone metastases only.

The analysis of BALP and CTX-1 levels was repeated after subgrouping patients by the presence of bone metastases only (with: N = 132, without: N = 47). The median baseline values recorded in the group with and without bone metastases were: 22.50 (range: 1–218) vs 15.90 (range: 1–162), respectively, for BALP (Fig. 5, top panel); 0.58 (range: 0.04–4.57) vs 0.52 (range: 0.17–2.19), respectively, for CTX-1 (Fig. 6, top panel). Over time, in both groups BALP progressively decreased down to similar levels starting from month 6, reaching statistical significance at month 12 vs baseline (P = 0.0043) in men with bone metastases only (Fig. 5, bottom panel). As for CTX-1, compared to the baseline levels, the values significantly decreased at month 6 in men with bone metastases (P = 0.0273), while they remained quite stable in men without bone metastases (Fig. 6, bottom panel).

## 5 Discussion

This ancillary analysis of ABITUDE is the first large prospective study testing, in a real-world setting, the effects of AAP on the levels of a panel of BTMs and bone-related markers in CT-naïve mCRPC patients after ADT failure. Overall, we observed fluctuations over time of all biomarkers: as the patients receiving AAP were already on treatment with LH-RH agonists, the fluctuations observed can be ascribed to AAP.

The progressive decrease of serum BALP levels over time may be indicative of an inhibitory effect on bone formation and of a reduction of disease extent. It has been shown that BALP levels usually increase during the first 2–6 weeks of treatment with AAP and later decrease in mCRPC patients who respond to treatment, likely as a result of tumor burden reduction and bone healing [34]. The initial increase is likely attributable to an increase of bone healing ad it is frequently observed in men with a sharp decline in PSA levels [16]. In accordance with these findings, Iuliani et al observed an increase in BALP levels in the serum of mCRPC men 3 months after the start of AAP therapy [35]. CTX-1, a marker of bone resorption, decreased up to month 6, in line with previous results from Iuliani et al, suggesting an anti-resorptive activity of AAP [35]. Importantly, the comparison between the levels of BALP and CTX-1 recorded in men with bone metastases vs those without bone metastases shed some light on the possible differential effects of AAP on metastatic vs normal bone. First, in both cases, we observed a decrease in the levels of BALP, that seemed more pronounced in men with bone metastases, likely due not only to the inhibitory effect on tumor growth but also to the interference with bone microenvironment at the level of metastatic site. As for CTX-1, the different trend observed between the two groups suggests that AAP is able to decrease bone resorption preferentially in the metastatic bone and not in normal bone. Of consequence, in all patients, it is unlikely that AAP increases fragility in non-metastatic bone, while it exerts a positive effect on bone metastases and their microenvironment. This is relevant in the setting of mCRPC, even in consideration of the fact that ADT promotes a rapid and dramatic increase in bone turnover that results in bone loss and qualitative/microarchitectural damage [36].

BTTs include bisphosphonates (BPPs, e.g., zoledronic acid and alendronate) and denosumab and are employed to attenuate cancer treatment-induced bone loss. A post-hoc analysis of COU-AA-302 has shown that adding BTT to AAP in patients with mCRPC and bone metastases improves the clinical benefit of the latter in terms of overall survival, time to ECOG deterioration and time to opiate use for cancer-related pain, while being safe and tolerated [37]. Moreover, in the ERA-223 trial, adding BTT in patients with CT-naïve asymptomatic o paucisymptomatic mCRPC on AAP and randomized to receive radium 223 (Ra223) or placebo decreased the rate of those with osteoporotic fractures in both arms (Ra223 arm: from 37% without BTTs to 15% with BTTs; placebo arm: from 15% to 7%, respectively) [38]. The role of BTTs in the treatment of mCRPC patients has been confirmed in the EORTC 1333/PEACE III trial, comparing enzalutamide and Ra223 versus enzalutamide alone in asymptomatic or mildly symptomatic mCRPC patients, in which the use of BTTs was made mandatory after the unblinding of ERA-223. Results showed that the risk of fractures is very well controlled in both arms, being almost abolished by the mandatory continuous BTT administration starting at least 6 weeks before the first injection of Ra223 [39]. However, in the real world, a considerable proportion of patients is not adequately treated to prevent SREs or manage pain [40], [41], [42] and our findings further support this evidence, with only 11.8% of mCRPC patients receiving BTT. Moreover, 3.8% of patients included in this ancillary analysis received calcium supplementation, and 14.0% vitD supplementation: the last EAU guidelines recommend offering these supplements when prescribing either denosumab or BPPs [43]. Based on the decrease in CTX-1 levels that we observed in mCRCP patients with bone metastases, it is possible to hypothesize a synergistic effect of AAP and BTTs in these setting. This observation deserves further confirmation.

The following limitations must be acknowledged: lack of a control group, limited sample size overall and of the actual number of patients without bone metastases (the rate, however, is in line with previous data [6]), and the number of drop-outs, which has been addressed by comparing at each time point only the samples obtained at baseline from the same patients (i.e., median intra-patient change). The main strengths of this analysis are that it is the first prospective study investigating the effect of AAP on BTMs and in a real-world setting, and that we distinguished BTT-untreated patients, thus avoiding the bias on bone turnover caused by exposure to these agents.

## 6 Conclusions

This is the first study evaluating BALP, CTX-1, PTH and vitD in mCRPC patients during a novel hormone therapy. The ancillary bone turnover analysis of the ABITUDE study demonstrates that AAP is able to positively interfere with bone turnover in the microenvironment of cancer metastases, while the effect on bone turnover of normal bone seems to be limited and not clinically significant. This activity likely contributes to AAP antitumoral effect.

## Funding

This study was funded by Janssen-Cilag SpA.

## CRediT authorship contribution statement

**Daniele Santini:** Conceptualization, Investigation, Resources, Writing - original draft, Writing - review & editing, Visualization, Supervision. **Saverio Cinieri:** Investigation, Resources, Writing - review & editing. **Donatello Gasparro:** Investigation, Resources, Writing - review & editing. **Roberto Bordonaro:** Investigation, Resources, Writing - review & editing. **Pamela Francesca Guglielmini:** Investigation, Resources, Writing - review & editing. **Vincenzo Emanuele Chiuri:** Investigation, Resources, Writing - review & editing. **Rolando M D’Angelillo:** Investigation, Resources, Writing - review & editing. **Giovanni Luca Ceresoli:** Investigation, Resources, Writing - review & editing. **Daniele Fagnani:** Investigation, Resources, Writing - review & editing. **Mirko Acquati:** Investigation, Resources, Writing - review & editing. **Manlio Mencoboni:** Investigation, Resources, Writing - review & editing. **Gaetano Lanzetta:** Investigation, Resources, Writing - review & editing. **Donata Sartori:** Investigation, Resources, Writing - review & editing. **Paolo Carlini:** Investigation, Resources, Writing - review & editing. **Fabiana Panebianco:** Writing - review & editing, Project administration. **Patrizia Beccaglia:** Writing - review & editing, Project administration. **Giuseppe Procopio:** Conceptualization, Investigation, Resources, Writing - original draft, Writing - review & editing, Visualization, Supervision.

## Declaration of Competing Interest

The authors declare the following financial interests/personal relationships which may be considered as potential competing interests: R. Bordonaro discloses honoraria/consulting or advisory role/speaker’s bureau from Bayer, AstraZeneca, Sanofi, Novartis, Amgen, Roche, Pfizer, Janssen Cilag, Bristol Mayer Squibb; travel accommodations expenses from Bayer, Pfizer, Astellas, Roche; fellowship or research program from AstraZeneca, Astellas. V.E. Chiuri discloses fees for Advisory Board and speaker from Bristol Mayer Squibb, Ipsen, Janssen Cilag and Pfizer. P. Carlini discloses congressional sponsorships from Sanofi, Aventis, Bayer SPA, Astellas, Janssen Cilag. F. Panebianco and P. Beccaglia are Janssen Cilag employees. G. Procopio discloses consultant or advisory board from AstraZeneca, Bayer, Bristol Mayer Squibb, Ipsen, Janssen Cilag, Merk, MSD, Pfizer, Novartis. The other authors have nothing to disclose.
